# Molecular Imprinting and Functional Polymers for All Transducers and Applications

**DOI:** 10.3390/s18020327

**Published:** 2018-01-24

**Authors:** Franz L. Dickert

**Affiliations:** Department of Analytical Chemistry, University of Vienna, Währinger Str. 38, A 1090 Vienna, Austria; Franz.Dickert@univie.ac.at; Tel.: +43-1-4277-52301

**Keywords:** molecular imprinting, transducers, aldehydes, phenoles, proteins, cells

## Abstract

The main challenge in developing a chemical sensor is the synthesis of recognition coatings, which are very sensitive and selective to analytes of interest. Molecular imprinting has proven to be the most innovative strategy for this purpose in functional polymer design in the last few decades. Moreover, the introduction of functional groups brings about new applications for all available transducers. Sensitivity and selectivity features of sensor coatings can be tuned by this approach. The strategy produces molecular cavities and interaction sites in sensor coatings. The synthesis of these tailored recognition materials is performed in an outstanding manner, saving time and the high costs of chemicals. Furthermore, intermolecular interactions between the analyte and chemical layers will generate sites that are complementary to the analyte. This procedure can easily be done, directly on a transducer surface, which entails engulfing the analyte by a prepolymer and crosslinking the polymeric material. These imprinted polymers form a robust recognition layer on the transducer surface, which cannot be peeled off and can withstand very harsh conditions, both in gaseous and liquid media. These recognition materials are very suitable, for small molecules and even large bioparticles.

## 1. Introduction

Approximately thirty years ago the Nobel Prize was awarded to J.-M. Lehn, D. Cram, and C. Pedersen for the design of supramolecular chemistry. This idea can be appreciated as an especially significant contribution to chemistry in the twentieth century. Thus, molecular recognition was introduced in chemistry in an innovative way. Furthermore, these receptors were synthesized via self-organizing processes. Hosts and guests adapt to each other, yielding an optimized fit between them. These molecular recognition methods are applicable both for ions and neutral molecules. 

These ideas were further developed with respect to polymers, which are more robust than molecules. Molecular imprinting was preferably stimulated by the ideas of G. Wulff [1] and K. Mosbach [2] approximately three decades ago. In this way, molecularly imprinted polymers (MIPs; NIPs—without template) that imitate nature by biomimetic strategies were designed and synthesized. This had such advantages as straightforward synthesis based on a large variety of cheap, easily accessible starting materials and no time-consuming efforts. The precursors engulf the analyte and form a highly cross-linked polymer. This is reached by adding an appreciable amount of bifunctional polymer precursors. 

These polymers can be combined with nearly all types of transducers, both for gaseous and liquid phases. The robust materials are protected against flooding of the sensor or dissolving of the coatings in liquid phases. Thus, the sensitive layers can be attached to mass-sensitive devices, interdigital electrodes, optrodes, or electrochemical electrodes. The universal applicability of the quartz-crystal-microbalance (QCM) has to be emphasized, which is also discussed in some of the papers cited. This QCM transducer shows that all rigidly adsorbed or absorbed analytes will give a frequency response. The basic quartz sheets of the QCMs can be bought for less than a dollar in the international market, and the fabrication of gold electrodes is easily possible via screen printing.

## 2. Summary of the Special Issue

### 2.1. Lean Molecules

Formaldehyde is widely used in chemistry, especially for resins and paints. It is a very reactive ingredient in production processes. The fabrication of chipboards is an extraordinary field of application of formaldehyde. Furthermore, it is used for disinfection. There are distinct rules concerning degassing of formaldehyde out of different synthetic products. The World Health Organization (WHO) and the National Institute limit the long-term exposure to formaldehyde to 0.08 and 1 ppm, respectively. This is a challenge for the indoor monitoring of this hazardous compound. 

This task [3] was envisaged combining MIPs as coating with QCMs. These transducers show a resonance frequency of 10 MHz, which guarantees high sensitivity and easy handling. Dual screen-printed electrodes were used for compensation of temperature fluctuations and other interferences. The detection of such a small molecule is no easy task since a minor mass yields only minor frequency responses. Furthermore, ambient humidity in air will lead to cross sensitivities since formaldehyde shows polar properties. This has to be thoroughly considered when the appropriate sensitive materials are designed. 

MIP syntheses were performed with different precursors such as styrene, methacrylic acid, and ethylene glycol dimethacrylate (EGDMA). An allyl amine was sometimes added. Radical polymerization was initiated by azobisisobutyronitrile (AIBN). Oligomers were used to coat the electrodes with MIPs and NIPs. Furthermore, the oligomers were pipetted in acetonitrile under vigorous stirring. This yields nanoparticles in an adequate size. Advantageous results were obtained with a copolymer of styrene. A limiting concentration of 1 ppm formaldehyde in air was observed. Problems arise in the presence of humidity. Significant responses vanish in this case. Adding an ally amine as a precursor for the formation of the polymer solves this problem. The underlying idea is based on the strong interaction between primary amines with the aldehyde group. Further improvements were made with the application of nanoparticles, as the nature of their surface leads to shortened response times. The required sensitivity of 0.5 ppm was attained. These innovative coatings showed a minor cross-sensitivity to dichloromethane, methanol, formic acid, acetone, ethanol, acetaldehyde, and acetonitrile. An appreciable difference by a factor of nearly 10 was found by comparing MIPs and NIPs.

Formaldehyde was monitored with an MIP consisting of polypyrrole as a sensitive material in another study [4]. Titanium dioxide nanotubes were used as support to increase the surface of the recognition layer. The nanotubes were vertically assembled on a titanium sheet and annealed at 475 °C. Polypyrrole gives rise to the possibility of generating the sensitive layer by electropolymerization. Polymerization took place in acetonitrile in the presence of the template formaldehyde. The titanium sheet acted as a working electrode, whereas platinum and Ag/AgCl acted as counter and reference electrodes, respectively. The template molecules were removed by acetic acid. The same process was performed without the addition of formaldehyde, which led to NIPs. Thus, the efficiency of the imprinting process was tested. Two gold electrodes on the device were used to perform conductivity measurements. Additional, pyrrole was polymerized on the gold electrodes of a QCM. Thus, the mass of adhered formaldehyde could be measured in an independent way.

The sensor materials were characterized with different methods such as SEM, which visualizes the dimensions of the nanotubes. FT-IR spectroscopy allows analysis of both MIP and NIP coatings. The C=O band characterizes the included template. Furthermore, energy dispersive X-ray spectroscopy (EDX) was used to evaluate the distinct elements of the coating and the substrate. The amount of formaldehyde absorption was directly monitored by QCM measurements. The responses of the NIP layers are nearly negligible to those of MIPs. The cross-sensitivities to acetaldehyde, acetic acid, ethanol, and acetone can be considered as more minimal. Minor influences of humidity were confirmed. Excellent sensor responses to formaldehyde were observed, which indicates that detection far below 1 ppm is possible. 

The next paper [5] discusses the detection of several types of aldehydes. Hexanal, nonanal, and benzaldehyde were chosen as analytes. These volatile aldehydes act as biomarkers for lung cancer. Again, MIPs are used to design recognition centers for these analytes. Molecularly imprinted sol–gel materials were chosen as sensor coatings. These materials have mild preparation conditions, are easily modified with organic groups, and have high thermal stability. Thus, these materials allow for the design of sensor arrays, which enables electronic selectivity via pattern recognition. Their syntheses were based on silanes and respective Ti compounds. The sensing materials were applied to 9 MHz QCMs and dried at 130 °C. The purity of the aldehyde vapors were checked by GC-MS analysis. In total, 14 different sensors were used with modified materials.

The sensors give remarkable frequency answers to different aldehydes. The success of imprinting is proven by the fact that MIP responses always appreciably exceed those of NIPs. The high selectivity of the sensor system can be quickly inferred from the radar chart and the score plot of principal component analysis. Furthermore, the quality of the different sensors were statistically characterized in terms of selectivity, frequency responses, and sensor dynamics. Furthermore, hierarchical clustering analysis was performed and is presented in a dendogram. Thus, a sensor array was designed without redundant channels. The results show that the developed sensor materials can be used for a selective pre-concentration of analytes. Beyond this, the sensor array can directly characterize complex matrices such as health status.

Bisphenol A is a widespread compound which is used for the production of epoxy resins and other polymers. Furthermore, it has been well known for many decades that bisphenol A mimics estradiol. Thus, it is a large threat for waste water, which can lead to a disturbance of the hormonal balance. Molecular imprinting was used again to create selective binding sites [6]. Furthermore, a diazomethane treatment was performed to improve selectivity. The detection method was based on capillary electrophoresis (CE) with ultraviolet monitoring. The MIPs were made from the precursors methacrylic acid and ethylene glycol dimethacrylate as cross linkers, with bisphenol A as a template. The MIP and NIP particles were treated with diazomethane to form an ester. In this way, the selectivity was improved. The extraction capabilities of MIP and NIP particles, esterified or not, were tested with CE-UV analysis. Additional mechanistic insights were obtained with high-performance liquid chromatography with UV detection. The selectivity of binding bisphenol A, diclofenac sodium salt, metformin hydrochloride, and baclofen to the MIP particles was studied. The ratio of template molecules to the polymer precursors were systematically varied to optimize the receptor sites. A high selectivity to bisphenol A was achieved in comparison to the interfering compounds. Interesting mechanistic insights were obtained by the diazomethane treatments. Thus, the differences between MIPs und NIPs were appreciable.

### 2.2. Proteins and Cells

There are many molecular imprinting papers with lean templates in comparison to bio-macromolecules or whole bio-particles. Here, only surface patterning can be done since diffusion processes in bulk are hindered by large particles. In one study [7], an electrode was used as a transducer. This implies that the polymer can be directly generated on the electrode by electropolymerization. A hexameric heme protein was chosen as a template. The polymer on the gold electrode was generated by scopoletin, which was electrochemically polymerized. The thickness of the polymer in which the template is embedded correlates directly to current and time. The template was oriented in an optimized way by applying a thiol layer to the gold electrode. The finished electrode was tested by cyclic voltammograms in respect to functionality. A positively charged redox marker, namely [Ru(NH_3_)_6_]^2+^, was used for this purpose. In parallel to modifications of the electrode by thiol, polymerized scopoletin, and adhered heme protein, a decrease in current is observed. The rebinding of hexameric heme protein was followed by cyclic voltammograms, which indicates the presence of the Fe^2+^/Fe^3+^ redox couple. The properties of the oxidized and reduced states of the protein are very similar. Thus, the change in the redox state does not lead to a change in binding efficiency. Appreciable differences can be observed when binding the template to MIPs and NIPs, which validates the imprinting strategy. Furthermore, the cross-sensitivity to cyctochrome c was tested, and minor adhesion to the imprinted electrode was shown. The catalytic reduction of hydrogen peroxide by the electrode-incorporated heme protein was also studied. Thus, surface imprinting is a trendsetting procedure for selectively accumulating proteins on surfaces.

General procedures of imprinting are discussed so as to optimize applications [8]. The goal is to transfer imprinting strategies to the market, and some observations are described. For example, increasing selectivity and affinity leads to problems with reversibility and response times. These difficulties must be solved in designing sensitive coatings for extended particles such as proteins or cells. The horizon is opened when surface imprinting is introduced, since diffusion is not hindered in this case. Thus, softlithography, e.g., stamping methods, reveals innovative perspectives especially with respect to bioanalytes. Furthermore, a sensor adapted to a distinct analyte must be combined with a suitable transducer. Mass-sensitive devices have the general advantage that every adhered analyte will give a frequency response. Ten megahertz QCMs were used. Mass-sensitive devices with a higher operating frequency allow for higher sensitivity. QCMs that have a grid electrode and no plane gold cover were applied. This revealed the possibility of detecting dielectric phenomena beyond the pure mass response. Polyurethanes were used in the printing process. A prepolymer was cast on the QCM sheet followed by cross-linking these materials. The cells were pressed in the resin before it became rigid. The QCM responses greatly differed for viable and non-viable cells. These findings are based on several effects, including capacitive or mechanical phenomena. Thus, it can be concluded that the transducer can reveal completely new possibilities in terms of differentiating between cells as reported for the grid electrodes. The measurements were only possible by molding the polymer to create hollows for cell engulfing. The method reported depends on the availability of reproducible templates. Furthermore, it was propagated to synthesize imprinted nanoparticles in water using an automated chemical reactor. This reveals the production of MIP receptors on an industrial scale so as to gain access to the market.

Erythrocytes show an outstanding importance for the body. Their sizes are approximately 6–8 μm. Oxygen transport in blood guarantees the survival of the organisms. Safe blood transfusion is only possible due to the pioneering work of Landsteiner, who elucidated the ABO blood group system. Today, it is standard procedure to determine human blood groups: A (containing only A antigens), B (containing only B antigens), AB (having both A and B antigens), O (neither A nor B antigens), and Rh (giving information about the presence or absence of Rh antigens). This is the basis for successful and safe blood transfusion. An incompatible transfusion would lead to agglutination, which implies a sudden death.

The classical methods blood group typing use the agglutination of blood. Different arrangements are suitable for this purpose, such as the slide method, tube tests, microplate technology, and column/gel centrifugation. They differ in their sensitivity, speed, and simplicity. An economic challenge is to envisage synthetic antibodies [9] that can recognize the antigens of erythrocytes. Again, no usual bulk imprinting can be performed since the erythrocytes have a diameter of 6–8 μm. Thus, surface imprinting must be performed. It has to be considered that erythrocytes have a flexible shape. Otherwise, they would not be able to migrate in thin capillaries of the body. The basis of imprinting is therefore reduced to interactions between the cell membrane and the surface of sensor coating. The shape of the imprint is not important. Suitable polymers are polyurethanes or polyvinylpyrrolidones. They can be combined with, e.g., QCMs or interdigital transducers for capacitive or resistive measurements. Blood cells are assembled on a pre-polymer followed by its hardening. The sensor responses are always most prominent when the template and analyte are identical. Again, the measurements are performed in a differential mode to eliminate unselective background effects. Sub-blood groups, such as A1 or A2, can be quantitatively determined and differ only by the amount of antigens.

## 3. Conclusions

Therefore, it can be concluded that imprinting is a powerful strategy to design and fabricate sensor coatings with selective recognition capabilities. A high variety of transducers can be combined with these coatings. Thus, this field of pure and applied science will further expand.
